# Mineralization Reduces the Toxicity and Improves Stability and Protective Immune Response Induced by *Toxoplasma gondii*

**DOI:** 10.3390/vaccines12010035

**Published:** 2023-12-28

**Authors:** Ling Li, Yong-Chao Guan, Shao-Yuan Bai, Qi-Wang Jin, Jian-Ping Tao, Guo-Ding Zhu, Si-Yang Huang

**Affiliations:** 1 Institute of Comparative Medicine, College of Veterinary Medicine, Yangzhou University, Jiangsu Co-Innovation Center for Prevention and Control of Important Animal Infectious Diseases and Zoonosis, Jiangsu Key Laboratory of Zoonosis, Yangzhou 225009, China; liviastark@163.com (L.L.); guanyongchao12@163.com (Y.-C.G.); shaoyuan2023@163.com (S.-Y.B.); qiwangj@163.com (Q.-W.J.); jptao@yzu.edu.cn (J.-P.T.); 2 Joint International Research Laboratory of Agriculture and Agri-Product Safety, the Ministry of Education of China, Yangzhou University, Yangzhou 225009, China; 3 National Health Commission Key Laboratory of Parasitic Disease Control and Prevention, Jiangsu Provincial Key Laboratory on Parasite and Vector Control Technology, Jiangsu Provincial Medical Key Laboratory, Jiangsu Institute of Parasitic Diseases, Wuxi 214064, China; jipdzhu@hotmail.com

**Keywords:** thermostability, immunization, protection, vaccine, toxoplasmosis

## Abstract

Vaccination is an ideal strategy for the control and prevention of toxoplasmosis. However, the thermostability and effectiveness of vaccines limit their application. Here, calcium mineralization was used to fabricate *Toxoplasma gondii* tachyzoites as immunogenic core–shell particles with improved immune response and thermostability. In the current study, *T. gondii* RH particles coated with mineralized shells were fabricated by calcium mineralization. The mineralized shells could maintain the *T. gondii* tachyzoites structural integrity for at least 12 months and weaken the virulence. Immunization of mice with mineralized tachyzoites induced high levels of *T. gondii-specific* antibodies and cytokines. The immunized mice were protected with a 100% survival rate in acute and chronic infection, and brain cyst burdens were significantly reduced. This study reported for the first time the strategy of calcium mineralization on *T. gondii* and proved that mineralized tachyzoites could play an immune protective role, thus expanding the application of biomineralization in *T. gondii* vaccine delivery.

## 1. Introduction

*Toxoplasma gondii* is one of the most important zoonotic pathogens in apicomplexan parasites and can infect almost all warm-blooded animals [1]. About one-third of the world’s population has been chronically infected [2]. *T. gondii* usually causes asymptomatic or subclinical infections in healthy people and could cause serious complications and even death in immunocompromised people and pregnant women [3]. Infection of pregnant women or pregnant animals may cause abortion, malformation and even stillbirth [4]. Sulfadiazine and pyrimethamine are only sufficient for acute infections, while no drug was available for chronic infection. The emergence of drug-resistant strains further limits the use of drugs [5].

Vaccination is one of the most significant achievements for controlling and preventing infectious diseases; it is also an ideal strategy for toxoplasmosis [6,7]. Current studies on the *T. gondii* vaccine mainly focused on killed vaccines, live attenuated vaccines [8], specific component vaccines [9,10], subunit vaccines [11] and nucleic acid vaccines [12]. Immunoprotection is still an important challenge for the *T. gondii* vaccine. Therefore, improving protection efficiency has become an urgent problem. ToxoVax, the only commercial attenuated vaccine of *T. gondii*, provides good protection against *T. gondii* infection among goats and sheep [13], while harsh storage and transportation conditions limit its use on a larger scale in the world [14]. Vaccines have strict requirements for storage and transportation conditions, which, if not met, decreases their effectiveness. How to improve the stability of vaccines has become an urgent problem.

Biomineralization is a universal natural phenomenon that helps organisms withstand harsh environments [15,16,17]. Previous studies showed that the self-mineralized human EV71 thermostable vaccine could be stored at 37 °C for at least seven days [18]. However, most living organisms are unable to generate mineralized shells without additional intervention, as the natural environment may lack sufficient inorganic or organic matter to produce mineralized shells. Biomineralization requires specific treatments and occurs under relatively mild conditions [4]. Studies on biomineralization showed that mineralized shells enhanced the stability of the virus vaccine, helped the vaccine induce a higher immune protection response, and reduced the pathogenicity of the virus [19]. However, mineralization studies on *T. gondii* have not been reported.

Thus, in this study, a novel attempt to encapsulate the *T. gondii* tachyzoites within calcium mineralization is presented. The aim of this study was to evaluate the immunogenic effect of the core–shell tachyzoites particles in a vaccine delivery for induction of immunity responses and identify the immune protection in mice. At the same time, we also clarified the thermostability of the core–shell tachyzoites particles.

## 2. Materials and Methods

### 2.1. Mice and Parasites

Seven-week-old SPF ICR mice were obtained from the Comparative Medical Centre of Yangzhou University. The animal study protocol was approved by the Institutional Animal Care and Use Committee (IACUC) of Yangzhou University (permit no. 202302012, 4 March 2023). Vero cells were cultured in 25 cm^2^ culture flasks (Thermo Scientific™, Waltham, MA, USA) with Dulbecco’s modified Eagle’s medium (DMEM, Gibco^TM^, Carlsbad, CA, USA) supplemented with 5% FBS, 100 U/mL penicillin, and 10 mg/mL streptomycin at 37 °C in a 5% CO_2_ atmosphere. Tachyzoites of the RH strain and PRU strain were cultured in monolayers of Vero cells in DMEM [20]. To harvest tachyzoites, cell debris was removed by a 3 µm pore membrane filter (Whatman, MA, USA). Tachyzoites were quantified using a hemocytometer before the next experiments.

### 2.2. Regulation and Determination of Tachyzoites ζ Potential

Before mineralizing tachyzoites, the membrane ζ potential must be measured. The tachyzoites’ surface charges could be regulated by changing the pH of the solution. Solutions with different pH values were prepared (pH 4–pH 10) and the tachyzoites were incubated in different pH solutions for 5 min before the ζ potential was measured by Malvern ζ sizer Nano ZS90 (Malvern Panalytical, Malvern, UK).

### 2.3. Mineralization of Tachyzoites

To screen the best coating condition, three biomineralizing conditions were used to prepare mineralized tachyzoites, as follows:

Method 1: 1 mL phosphate-buffered saline (PBS, PH7.5) with 2.4 × 10^6^ RH tachyzoites was mixed with 1.1 mL Ca(NO_3_)_2_ (30 mM) and mixed upside down at 4 °C for 3 h, after which 7.5 mL (NH_4_)_2_HPO_4_ (5 mM) was added to the above solution and mixed upside down at 4 °C for 45 min. The biomineralized product was named Mineralized tachyzoites 1.

Method 2: 1 mL PBS with 2.4 × 10^6^ RH tachyzoites were mixed with 1.1 mL Ca(NO_3_)_2_ (50 mM) and mixed upside down at 4 °C for 3 h, after which 7.5 mL (NH_4_)_2_HPO_4_ (5 mM) was added to the above solution and mixed upside down at 4 °C for 45 min. The biomineralized product was named Mineralized tachyzoites 2.

Method 3: 1 mL PBS with 2.4 × 10^6^ RH tachyzoites was mixed with 1.1 mL Ca(NO_3_)_2_ (30 mM) and mixed upside down at 4 °C for 3 h. Then, 7.5 mL (NH_4_)_2_HPO_4_ (20 mM) was added to the above solution and mixed upside down at 4 °C for 45 min. The biomineralized product was named Mineralized tachyzoites 3.

Mineralized tachyzoites were separated by centrifugation at 350× *g* for 10 min, the supernatant was moved to a new tube and the pellet was resuspended with PBS, and the mineralization efficacy (ME) was calculated by calculating the amount of unmineralized tachyzoites in the supernatant relative to the total amount of tachyzoites in samples as previous study [21].

$$\mathrm{ME}\%=(\;1-\frac{Amount\;of\;unmineralized\;tachyzoites\;in\;supernatant}{Total\;amount\;of\;tachyzoites\;in\;samples})\times 100\%$$

### 2.4. Scanning Electron Microscopy Observation

Mineralized tachyzoites were fixed overnight with 2.5% glutaraldehyde at 4 °C. The precipitate was separated by centrifugation at 350× *g* for 10 min, and then washed twice with PBS. After gradient dehydration with 30%, 50%, 70%, 80%, 90%, 95%, and 100% ethanol, the critical point drying was carried out, and the mineralized tachyzoites were sprayed with gold and observed with scanning electron microscopy (SEM) [22].

### 2.5. Thermostability Test

To evaluate the stability of mineralized tachyzoites, mineralized tachyzoites and unmineralized tachyzoites were kept in DMEM at 4 °C and 25 °C, respectively. Samples were taken to examine the stability at 3, 6 and 12 months. The samples were centrifugated at 700× *g* for 10 min and washed with PBS three time, then genomic DNA of tachyzoite precipitates from different groups were extracted, respectively. B1 gene of *T. gondii* was amplified from these samples by nested PCR.

### 2.6. Evaluation of Mineralized Tachyzoites Safety in Mice

To evaluate mineralized tachyzoite safety as a vaccine, 40 mice were randomly and evenly divided into four groups (10 mice per group). The mice of groups I to III were intraperitoneally injected with 1 × 10^5^, 1 × 10^6^ or 1 × 10^7^ mineralized tachyzoites, respectively, and group IV was intraperitoneally injected with 1 × 10^3^ unmineralized RH tachyzoites as a control. After injection, the survival of all mice was recorded daily for 30 days.

To further evaluate the biosafety of mineralized tachyzoites, the proliferation ability of mineralized tachyzoites was evaluated in mice. Five mice were intraperitoneally injected with 1 × 10^7^ mineralized tachyzoites as the first-generation infected group. After 14 days, another 5 mice were intraperitoneally injected with 0.5 mL peritoneal solution from the first-generation mice as the second-generation infected group, and 14 days later, another 5 mice were intraperitoneally injected with 0.5 mL peritoneal solution from the second-generation mice as the third-generation infected group. Five mice were intraperitoneally injected with 1×10^3^ normal tachyzoites as a control group. The morbidity and mortality of mice was recorded daily during the experiment.

### 2.7. Vaccination of Mice

In this study, the efficacy of mineralized tachyzoites in conferring protective immunity was evaluated against acute and chronic *T. gondii* infections. Eighty mice were randomly divided into two groups (40 mice per group): the first group was immunized twice with 1 × 10^6^ mineralized tachyzoites suspended in 200 µL PBS, and the second group was mock-vaccinated in a total of 200 µL PBS i.p. as a control.

### 2.8. Antibody and Cytokine Measurement

To detect the specific antibodies (IgG, IgG1 and IgG2b), blood samples were collected from the jugular vein at 28 and 56 days post-immunization (dpi), the blood samples were kept at room temperature for two hours and then kept at 4 °C overnight. The sera were separated by centrifugation at 700× *g* for 10 min and stored at −20 °C. Serum samples from mock-vaccinated mice were used as negative controls. Meanwhile, 5 mice were sacrificed in each group, and the splenocytes were aseptically collected for cytokine measurements.

Total immunoglobulin G (IgG) content and subclasses of IgG antibodies IgG1 and IgG2b, as indicators of Th2 and Th1 responses were tested by ELISA, as described previously [23]. Splenocytes were stimulated with 10 μg/mL STAg and culture supernatants were collected for determination of IL-4, IL-5, IL-6, IL-10, IL-23 and IFN-γ by ELISA according to the manufacturer’s instructions (Mlbio, Shanghai, China).

### 2.9. Protection of Mice against Acute and Chronic Infection

Fifty-six days after immunization, to evaluate the protective effect against acute infection, 10 mice from each group were challenged i.p., with 200 μL PBS containing 1 × 10^3^ RH tachyzoites, and the survival rate of infected mice was recorded for 30 days. To evaluate the protection against chronic infection, another 10 mice from each group were challenged i.p., with 200 μL PBS containing 1 × 10^5^ PRU tachyzoites. The survival rate was recorded daily for 30 days. For chronic infection, the survival mice were euthanized at 30 dpi, and each brain of the mice was homogenized in 1 mL of PBS, 10 μL sample was used to count the number of cysts and calculated the amount of the parasite cyst burden in the brain as described previously [8].

### 2.10. Statistical Analyses

To evaluate the level of the differences in antibodies, cytokines, and parasite cyst burdens between experimental groups and control groups, statistical analysis was performed using a two-tailed, unpaired Student *t*-test and one-way ANOVA (for comparing means between ≥ three groups). The Mantel–Cox log-rank test was used for comparing the survival curves of the different mouse groups. A *p* < 0.05 was considered statistically significant.

## 3. Results

### 3.1. Regulation and Determination of Tachyzoites ζ Potential

**Figure 1:**
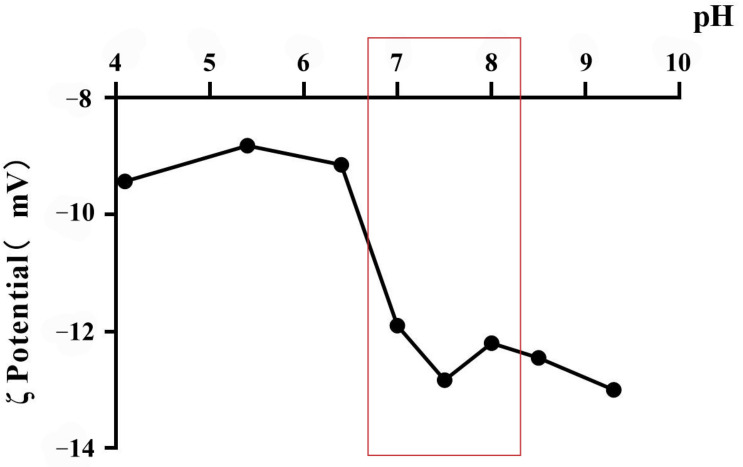
ζ potential on tachyzoites of *Toxoplasma gondii* RH strain under different pH conditions. The red box represents the candidate range.

Tachyzoites were incubated in different pH conditions, and then the ζ potential of tachyzoites was measured by Malvern ζ sizer Nano ZS90. From the results we found that tachyzoites carried negative charges under different pH conditions (pH 4–pH 10), and the surface potential of tachyzoites increased with the increase in pH (Figure 1). Based on the above results, and combined with physiological factors and mineralization conditions, we selected PBS pH 7.5 as the mineralization solution.

Tachyzoites of the *T. gondii* RH strain were mixed with different pH solutions, and the surface membrane potential of tachyzoites at different pH conditions was measured by Malvern particle analyzer.

### 3.2. Characterization of Mineralized Tachyzoites

**Figure 2:**
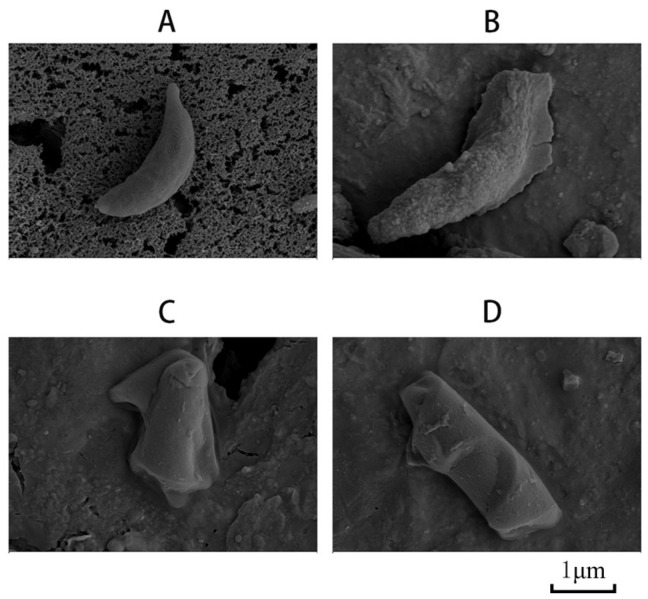
Characterization of biomineralized tachyzoites of the *Toxoplasma gondii* RH strain. (**A**) SEM images of tachyzoites of the *T. gondii* RH strain not treated. (**B**–**D**) SEM images of mineralized tachyzoites treated by Method 1, Method 2 and Method 3, respectively.

The mineralized tachyzoites treated with different conditions were analyzed by SEM, and the unmineralized tachyzoites were analyzed as a control. From the results, we found that the unmineralized tachyzoites had a normal crescent shape (Figure 2A). Compared with the unmineralized tachyzoites, the surface of the mineralized tachyzoites was evenly covered with a layer of mineral and maintained the original form of the tachyzoite in the mineralized tachyzoite 1 group (Figure 2B). In the other two groups, the tachyzoites were also covered with minerals, changing their form significantly because of over-wrapping, and the original form was completely invisible (Figure 2C,D). The results indicated that method 1 was the best reaction condition, so it was selected for further study.

The cell counting chamber was used to count the unmineralized tachyzoites in the supernatant after centrifugation, and the ME was calculated using the formula above; the result showed that the ME was 80% in the mineralized tachyzoite 1 group.

### 3.3. The Stability of Mineralized Tachyzoites

**Figure 3:**
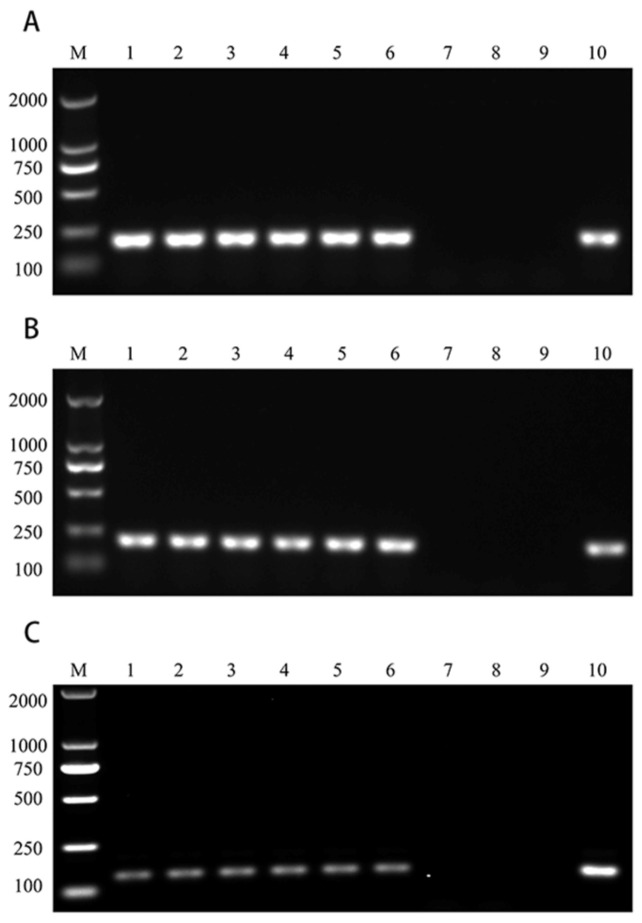
Mineralized tachyzoites were identified B1 gene with PCR under different preservation times. (**A**–**C**) PCR detection results of the B1 gene of mineralized tachyzoites and unmineralized tachyzoites preserved for 3, 6 and 12 months, respectively. (1–3) Mineralized tachyzoites preserved by DMEM at 25 °C; (4–6) Mineralized tachyzoites preserved by DMEM at 4 °C; (7–9) Unmineralized tachyzoites preserved by DMEM at 4 °C; (10) Positive control.

From the results, it can be concluded that the PCR results of mineralized tachyzoites stored at 4 °C and 25 °C for 3 months were positive, while those of unmineralized tachyzoites were negative (Figure 3A). The same results were obtained after 6 and 12 months of storage (Figure 3B,C). These results suggested that mineralized crusts can improve the stability of tachyzoites, allowing them to remain intact for a longer period of time, while unmineralized tachyzoites quickly disintegrated in one month. Even at 25 °C, the stability of mineralized tachyzoites was still very good.

### 3.4. Safety Evaluation of Mineralized Tachyzoites

**Figure 4:**
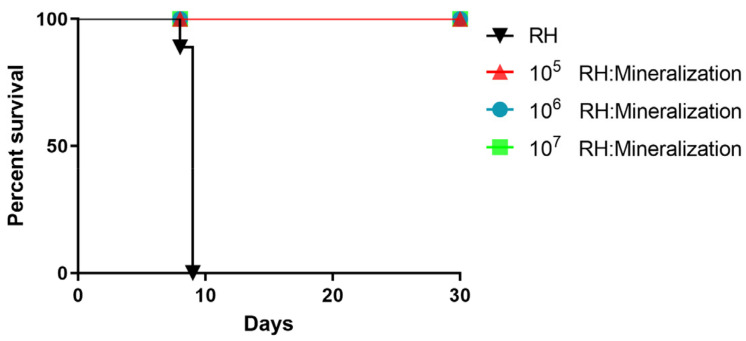
Safety evaluation of mineralized tachyzoites in mice. Survival curves of mice challenged intraperitoneally with 1 × 10^5^, 1 × 10^6^, 1 × 10^7^ mineralized tachyzoites, and 1 × 10^3^ unmineralized tachyzoites (as control), respectively. No mice were dead in mineralized tachyzoites groups.

To evaluate the safety of mineralized tachyzoites as a vaccine, we studied the effect of mineralization on the virulence of *T. gondii* in mice. From the results, we found that mineralization completely reduced the pathogenicity of the *T. gondii* RH strain. Mice infected with up to 1 × 10^7^ mineralized tachyzoites all survived without any clinical signs of toxoplasmosis. In contrast, mice infected with 1 × 10^3^ unmineralized tachyzoites became moribund and died within nine days post-infection (Figure 4). These results showed that mineralization significantly reduced the pathogenicity of *T. gondii*.

To further study the proliferation ability of mineralized tachyzoites in mice, ICR mice were infected with 1 × 10^7^ mineralized tachyzoites and blind transmission for three consecutive generations on mice. Our data indicated that the proliferation of mineralized tachyzoites had been completely lost, after passing for two or three generations in vivo; the mice had been not infected and continued to live healthily, while mice infected with 1 × 10^3^ unmineralized tachyzoites died within nine days post-infection (Table 1).

The result indicated that the virulence of mineralized tachyzoites had been lost compared with unmineralized tachyzoites, which was safe for a vaccine candidate.

### 3.5. Immune Response Induced by Mineralized Tachyzoites

**Figure 5:**
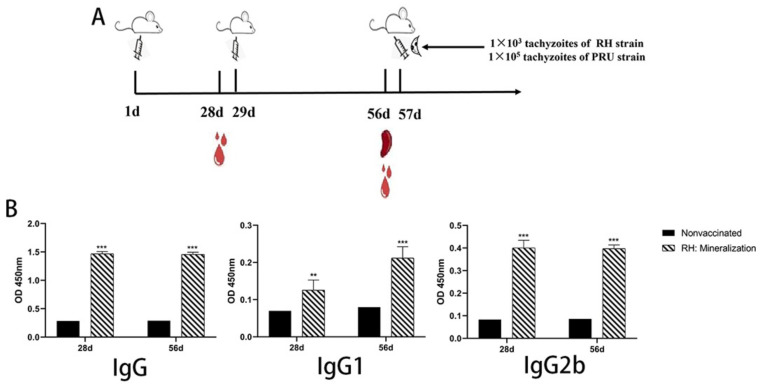
Serum antibody levels of mice immunized with mineralized tachyzoites. (**A**) Schematic depiction of mineralized tachyzoite immunoprotection scheme. (**B**) Blood samples were collected at 28 and 56 dpi to separate the serum. Total IgG antibodies and specific IgG isotype profiles were detected by ELISA. Results are expressed as the mean of OD450 ± standard deviation. Significance compared with control mice: ** *p* < 0.01. *** *p* < 0.001.

The immunogenicity of mineralized tachyzoites was assessed by examination of specific anti–*T. gondii* IgG antibody titers and IgG isotypes in the serum of immunized mice at 28 and 56 dpi. At day 28 after vaccination, all immunized mice had seroconverted with a higher level of specific *T. gondii* IgG antibodies compared with nonimmunized mice (Figure 5B). This level of IgG titer remained high at 56 dpi, which suggested that mineralized tachyzoites could induce a high-level humoral immune response in the immunized mice. The level of IgG2b was significantly higher in immunized mice at 28 and 56 dpi, while the level of IgG1 was highly increased after the second immunization compared to nonvaccinated mice. These results suggested that immunization with mineralized tachyzoites in mice could elicit a mixed Th1/Th2 immune response at 28 dpi, while the Th2 immune response was not as high as Th1; after the second immunization, the Th2 immune response increased.

**Figure 6:**
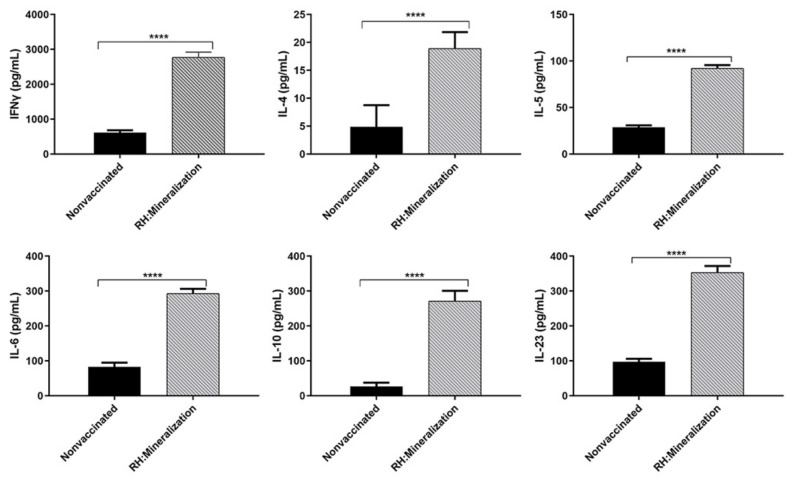
Cytokine levels in splenocyte culture supernatant of mice immunized with mineralized tachyzoites. **** *p* < 0.001 compared with control mice.

Spleen lymphocytes were isolated and stimulated with STAg for cytokine detection at 56 days after immunization, and cytokine expression in splenocyte culture supernatants was detected by ELISA. Levels of cytokines were significantly higher in the immunized mice compared with the unimmunized mice (Figure 6). All of these results indicated that mineralized tachyzoites could induce a mixed Th1/Th2 response in immunized mice.

At 56 dpi, splenocytes from 5 mice in each group were randomly collected and stimulated with 10 µg/mL STAg in vitro to collect cell culture supernatant for detection of cytokines. 

### 3.6. Protection against Acute and Chronic Infection

**Figure 7:**
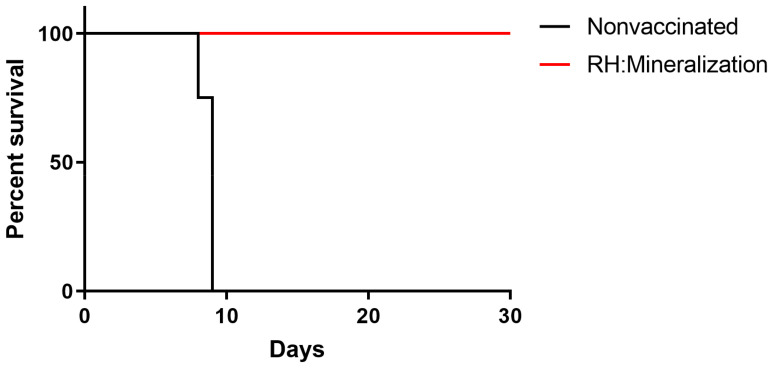
Protection of mice against acute *Toxoplasma gondii* infection.

Mice were immunized with 1 × 10^6^ mineralized tachyzoites, and 56 days later, challenged with 1 × 10^3^ tachyzoites of the *T. gondii* RH strain. As expected, all unimmunized mice challenged with 1 × 10^3^ RH tachyzoites died within 9 days, and the survival rate of mineralized tachyzoites vaccinated mice challenged with the *T. gondii* RH strain was 100% (Figure 7). The result showed that mineralized tachyzoites had a protective effect on acute infection.

**Figure 8:**
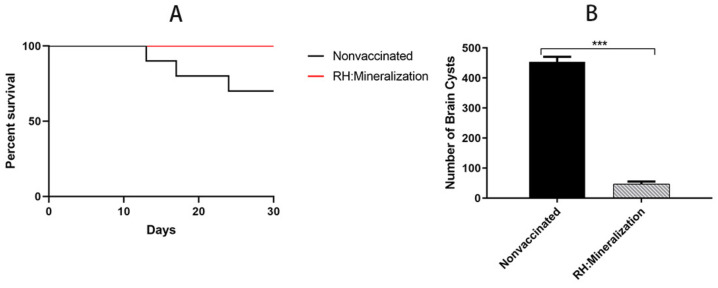
Protection of mice against chronic *Toxoplasma gondii* infection. (**A**) Survival curves of mineralized tachyzoites-vaccinated mice challenged with 1 × 10^5^ tachyzoites of PRU strain at 56 dpi (n = 10 mice). (**B**) Protection efficiency of mineralized tachyzoites against chronic infection was determined by cyst burden assays. Cyst burden in the brain of mice immunized with mineralized tachyzoites was significantly reduced (*** *p* < 0.001).

Survival rate and brain cyst burdens of mice were monitored to estimate whether mineralized tachyzoites could offer effective protection to mice against chronic infection of *T. gondii*. As expected, all immunized mice challenged with 1 × 10^5^ tachyzoites of the *T. gondii* PRU strain survived, whereas 30% of unimmunized mice died after challenged (Figure 8A). At 30 days post-challenge, parasite cyst burden in the brain of surviving vaccinated compared with nonvaccinated mice was determined. Unimmunized mice challenged with 1 × 10^5^ tachyzoites of the *T. gondii* PRU strain had 453 ± 30 cysts per brain, whereas mineralized tachyzoites immunized mice challenged with the same number of Pru tachyzoites had significantly fewer cysts per brain (45 ± 9 cysts/brain) (*p* < 0.01 Figure 8B). These results showed that mineralized tachyzoites provided excellent protection against chronic infection of *T. gondii*.

Survival curves of mineralized tachyzoites vaccinated mice challenged with 1 × 10^3^ tachyzoites of the RH strain at 56 dpi (*n* = 10 mice). Log-rank (Mantel–Cox) test was used for statistical analysis.

## 4. Discussion

We improved the immunogenicity and thermostability of *T. gondii* by mineralizing tachyzoites with CaP onto their surface. At the same time the virulence and proliferation of mineralized tachyzoites were lost. Based on these results, mineralized tachyzoites might be a promising vaccine to prevent toxoplasmosis. The current key problems for *T. gondii* vaccines were poor immunogenicity and thermostability, which limited their research [24,25,26]. In our study, calcium mineralization was used to improve *T. gondii* stability. Tachyzoites treated by mineralization also showed a good immune protection effect.

We first measured the surface potential of *T. gondii* RH tachyzoites, which carried negative charges in PBS (pH 7.5). *T. gondii* is different from virus and protein particles; its biological characteristics are more complex, so the mineralized reaction condition should be milder. The unsuitable acid-based environment causes damage to biological characteristics [27,28]. Under this condition, the surface of tachyzoites carried enough negative charges to absorb Ca^2+^ in the solution environment. SEM results showed that mineral shells had been formed on the surface of *T. gondii*. We chose the first synthesis method which could form mineralized shells without changing the morphology of tachyzoites.

Thermostability of vaccines is of great significance to the efficacy, preservation and transportation of the vaccines. The poor stability of the vaccine has severely affected the use and efficacy of the vaccine [29,30]. Biomineralization has been proven to help biomaterials respond to environmental changes [31]. We evaluated whether mineralized *T. gondii* tachyzoites were more stable than unmineralized tachyzoites. *T. gondii* tachyzoites died and were lysed after about 20 days at 4 °C [32]. The integrity of the mineralized tachyzoites stored for 3, 6 and 12 months was identified by PCR. These results showed that mineralized shells significantly improved the stability of tachyzoites. The thick mineral shell of mineralized tachyzoite extended its storage life at 4 °C or 25 °C by more than 12 months, longer than unmineralized tachyzoites, which could only be stored for 20 days.

Biosafety is an important prerequisite for vaccines [33,34,35]. We evaluated the virulence and proliferation of mineralized tachyzoites in mice. No mice died after intraperitoneal injection of 1 × 10^7^ mineralized tachyzoites, and blind transmission assay also indicated that tachyzoites had lost proliferation ability after mineralization. These results demonstrated the high safety of mineralized tachyzoites as vaccines.

No studies have yet demonstrated the immunogenicity of mineralized tachyzoites as a vaccine. In this study, we found that immunity induced by two vaccinations of mineralized tachyzoites completely protected all mice against a lethal challenge with *T. gondii* RH tachyzoites. Likewise, mineralized tachyzoites protected mice from chronic toxoplasmosis, and the cyst burden on brain tissue was significantly reduced. Consistent with other *T. gondii* vaccines, immunization with mineralized tachyzoites induced high levels of specific anti-*T. gondii* IgG antibodies. These specific antibodies block *T. gondii* invasion and attachment to host cells. and cleave extracellular tachyzoites in the complement pathway [36]. Cytokine assay showed that mineralized tachyzoites induced high levels of Th1-associated cytokines (IFN-γ). IFN-γ-dependent cell-mediated immune responses are necessary to limit *T. gondii* infections [37,38]. We also found increased expression of Th2-biased cytokines (IL-4, IL-6 and IL-10). They are important negative regulators of the inflammatory response and avoid host death due to excessive inflammatory response during *T. gondii* infection [39,40,41]. High levels of IL-5 promote the maturation of eosinophils and activate the ability to kill parasites [42]. Mineralized tachyzoites induced more IL-23, which induced NK cells and T cells to secrete more IFN-γ [43]. The above results provide evidence for the prevention of *T. gondii* infection by mineralized tachyzoites. In the future, mineralized tachyzoites will become candidate vaccines for small ruminants and other animals, including cats. This study provided technical and theoretical support for the prevention and control of toxoplasmosis.

## 5. Conclusions

The optimal thermostability and immunogenicity of mineralized tachyzoites favor their practical utilization. The safety of mineralized tachyzoites has been carefully evaluated, which is safer than attenuated vaccines, at the same time the protection is as effective as attenuated vaccines. The application of mineralized *T. gondii* vaccines with long-term stability not only reduces the cost of vaccine transportation and preservation, but also promotes the use of *T. gondii* vaccines on a large scale. The mineralization strategy of *T. gondii* paves the way for the development of high-performance *T. gondii* vaccines and is beneficial for clinical vaccine large-scale roll-out.

## Figures and Tables

**Table 1:** Morbidity and mortality of mice after continuous passage with mineralized tachyzoites.

Group	Number of Animals	Morbidity (%)	Mortality (%)	Survival Time (Days)
G1	5	0	0	>30
G2	5	0	0	>30
G3	5	0	0	>30
Control	5	100	100	8 ± 1

## Data Availability

Data are contained within the article.
